# Integration of prostate-specific membrane antigen-PET and multiparametric MRI for gross tumour volume definition in localised and locally advanced prostate cancer treated with image-guided radiotherapy

**DOI:** 10.1097/MOU.0000000000001321

**Published:** 2025-07-15

**Authors:** Tessa D. van Bergen, Arthur J.A.T. Braat, Cornelis A.T. van den Berg, Timo F.W. Soeterik

**Affiliations:** a Computational Imaging Group for MR Diagnostics and Therapy, Center for Image Sciences; b Department of Radiology and Nuclear Medicine, Division of Imaging and Oncology; c Department of Radiation Oncology, University Medical Center Utrecht, Utrecht, The Netherlands

**Keywords:** focal boosting, gross tumour volume, image-guided radiotherapy, MRI, prostate cancer, prostate-specific membrane antigen PET

## Abstract

**Purpose of review:**

This review evaluates recent evidence on the utility of multiparametric MRI (mpMRI), prostate-specific membrane antigen (PSMA) PET, and their combined application for accurately delineating the intraprostatic gross tumour volume (GTV) in patients with primary localised and locally advanced prostate cancer. It further explores the impact of GTV-based dose escalation on treatment-related toxicity and clinical outcomes.

**Recent findings:**

Recent studies suggest that combining PSMA-PET with mpMRI enhances lesion coverage of clinically significant, histopathologically verified intraprostatic tumours and yields higher interobserver agreement. However, this improved sensitivity is offset by reduced specificity, and it remains uncertain whether expanding the GTV to include additional PSMA-PET-defined regions impacts long-term treatment-related toxicity or improves oncological outcomes. Multiple phase I/II trials using PSMA-PET and mpMRI have reported acceptable acute and late toxicity profiles. Nevertheless, extensive data on long-term toxicity and disease outcomes following PSMA-PET-guided interventions remain limited, warranting further investigation to assess its impact.

**Summary:**

The combination of mpMRI and PSMA-PET has been shown to improve coverage of dominant intraprostatic lesion and reduce interobserver variability. While GTVs derived from combined imaging modalities are typically larger than those based on mpMRI alone, hypofractionated focal boost treatments targeting PSMA-PET/mpMRI-defined GTVs have demonstrated acceptable acute toxicity profiles. More data are needed to determine the impact of PSMA-PET expanded GTVs on long-term clinical outcomes.

## INTRODUCTION

A key treatment modality for the management of localised prostate cancer (PCa) is radiotherapy, which relies on precise targeting of the affected region. Higher radiation doses have been associated with a reduced risk of biochemical recurrence, potentially improving outcomes in patients at high risk of treatment failure [1]. However, escalating the dose to the entire prostate increases the risk of toxicity and treatment-related side effects [2,3]. Dose escalation up to 80 Gy EQD2 is generally considered effective and well tolerated when carefully planned and delivered [4].

Advanced techniques such as focal boost therapy aim to escalate radiation doses specifically to dominant intraprostatic lesions (DILs) while minimizing exposure to surrounding healthy tissue. The landmark FLAME trial showed that an integrated boost improved biochemical disease-free survival, without increasing toxicity or negatively impacting quality of life [4,5].

Accurate identification and delineation of the boost volume – commonly referred to as the gross tumour volume (GTV) – is a critical step in targeting the most aggressive intraprostatic tumour foci. Precise GTV definition enables optimised dose escalation to the DIL while sparing surrounding normal tissues, thereby reducing the risk of treatment-related toxicity. Delineation of intraprostatic lesions is primarily guided by advanced imaging modalities. Multiparametric MRI (mpMRI) is currently the most used technique for identifying index lesions and served as the imaging basis for target definition in the FLAME trial, as well as several other clinical studies investigating focal boost strategies [4,6,7]. However, studies have demonstrated that mpMRI may fail to detect all index lesions within the prostate [8,9^▪^]. Furthermore, substantial interobserver variability has been observed among radiation oncologists when contouring tumour targets on mpMRI alone for focal radiotherapy boost planning [10^▪^].

Prostate-specific membrane antigen PET (PSMA-PET) imaging has emerged as the standard of care for primary staging in patients with high-risk PCa [11]. Recent studies suggest that combining PSMA-PET with mpMRI may enhance the detection of clinically significant intraprostatic lesions [12,13^▪^,14^▪^]. Whether including additional PSMA-PET-defined regions in the GTV improves outcomes or affects toxicity remains uncertain. 

**Box 1:**
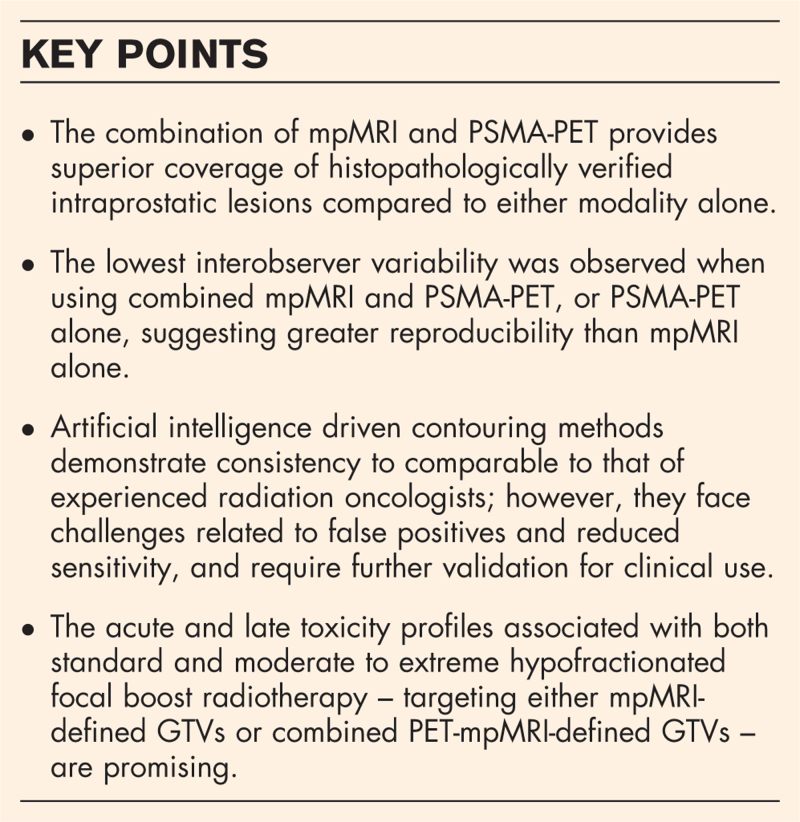
no caption available

## SEARCH STRATEGY AND SELECTION CRITERIA

The search strategy and selection criteria are described in supplementary file 1 and all results are presented as a narrative review (Supplementary File 1).

## PROSTATE-SPECIFIC MEMBRANE ANTIGEN-PET AND/OR MULTIPARAMETRIC MRI FOR INTRAPROSTATIC LESIONS CONTOURING

To ensure accurate GTV delineation, adequate assessment of the correlation between identified lesions on imaging and final histopathology is crucial. Several studies have compared imaging-defined targets with digitally annotated clinically significant PCa (csPCa) with whole-mount histopathology. This approach allows for volumetric comparisons and accurate validation of GTV delineation across imaging modalities.

### MRI-based gross tumour volume delineation and correlation with histopathology

Grefve *et al.* [15^▪^] analysed lesion coverage of single (T2w, DCE, or DWI) MRI modalities or mpMRI-based lesions, incorporating a clinical target volume margin of 0–3 mm (Table 1). Here, mpMRI provided greater specificity to the histopathological region compared to single MRI sequences, both with and without added margin. This was supported by better lesion coverage and Dice similarity coefficient (DSC), which measure the overlap between histopathological tumour region and segmented area.

**Table 1:** Accuracy of gross tumour volume delineation of intra-prostatic lesions using prostate-specific membrane antigen-PET and/or multiparametric MRI

Study	Imaging modality	Patient cohort	Histopathology reference	Best performing	Lesion specific outcome metrics	Mean sensitivity lesion detection	When reported: Median DSC; Lesion coverage; Lpath; HD_95%_; FDR
Geboers *et al.* [22^▪^]	PSMA-PET/CT (different tracers), mpMRI, combined	138 patients - 552 prostate quadrants	Quadrant-based analysis matched to whole mount histopthology	PSMA-PET + mpMRI	Detection rates of quadrants containing csPC	80%	N.A.
Grefve *et al.* [15^▪^]	[^68^Ga]-PSMA-PET/MR, mpMRI (and separate T2w, DWI, DCE), CTV-margin analysis, combined	15 high-risk PCa patients imaged preradical prostatectomy	Whole-mount histopathology via 3D-printed moulds.	[^68^Ga]Ga-PSMA-PET-11 + mpMRI	DSC, voxel-wise lesion coverage	N.A.	0.45 (DSC), 66% lesion coverage
Zarei *et al.* [19^▪▪^]	[^68^Ga]Ga-PSMA-11 PET/MR	15 high-risk PCa patients imaged preradical prostatectomy	Whole-mount histopathology (ISUP GG≥4 foci)	Manual delineation [^68^Ga]Ga-PSMA-11 PET/MRI	DSC, HD_95%_, lesion coverage ratio^a^, FDR	N.A.	0.44 (DSC); 7.58 mm (HD_95%_), 0.47% lesion coverage, 0.55 FDR
Zhang *et al.* [20]	[^68^Ga]Ga-PSMA-11 PET/MRI, [^18^F]F-PSMA-1007 PET/MRI, mpMRI	51 patients (40 [^18^F]F-PSMA-1007, 11 [^68^Ga]Ga-PSMA-11	Histopathological registration + correlation	No significant difference in tracer subgroups	Consistency of tumour length	N.A.	Lpath: 0.837 (ICC [^68^Ga]Ga-PSMA-11), 0.893 (ICC [^18^F]F-PSMA-1007)

DSC, Dice similarity coefficient; FDR, false discovery rate; GG, Grade Group; GTV, gross tumour volume; HD_95_^%^, 95^th^ percentile of the ordered Hausdorff distance; ICC, intra-class correlation coefficient; Lpath, consistency of tumour length; mpMRI, multiparametric MRI; PSMA-PET, prostate-specific membrane antigen PET.

a Lesion coverage ratio represents the proportion of histopathology lesion covered.

### Interobserver variability in MRI-based gross tumour volume delineation

Although key frameworks, such as the Prostate Imaging Reporting and Data System, have been developed to standardise image acquisition and interpretation of prostate MRI reading, substantial variability in MRI-based GTV delineation persists [16].

Jeganathan *et al.* [17^▪^] conducted a retrospective, single-centre study (*n* = 64) to quantify interobserver variability in DIL delineation across different mpMRI (T2w, b2000, ADC maps, DCE) sequences. The combined sequences resulted in the largest contours and had the highest DSC between observers. Lastly, lesion size and a PI-RADS (v2.1) classification of 5 were positively correlated with a higher DSC between observers.

Salgues *et al.* [18^▪^] conducted a similar study in a multicentre cohort (*n* = 68). They observed the mean distance to agreement (MDA), which measures the boundary error, to be lowest for the combined (T2+ADC+b2000+DCE) contours (4.2 mm). The combination of T2wADC had the highest median DSC (0.69), followed by T2w+ADC+DCE (0.67), and the combined contours (0.67).

Although interobserver variability remains prevalent, combined use of all sequences is recommended as it led to the most optimal GTV coverage and reduced interobserver variability.

### MRI- and PET-based gross tumour volume delineation and correlation with histopathology

Most studies delineating the GTV on PSMA-PET use manual segmentation or thresholding based on voxel-wise standardised uptake values (SUV), applying either absolute values or a fixed percentage of the maximum SUV (SUV_max_) within the prostate [19^▪▪^,^20^].

In a prospective study of 15 high-risk PCa patients undergoing [^68^Ga]Ga-PSMA-11 PET/MRI preradical prostatectomy with whole-mount histopathology, Zarei *et al.* [19^▪▪^] evaluated different GTV delineation methods (Table 1). Lower absolute thresholds increased lesion coverage and volume but did so at the expense of increased false discovery rate, reduced DSC, and greater boundary error. Manual contours made by four radiation oncologists were combined using the simultaneous truth and performance level estimation (STAPLE) algorithm to generate the most accurate tumour outline [19^▪▪^,^21^]. The resulting consensus contours demonstrated the highest concordance with histopathology, with a GTV-to-histology volume ratio of 1.08, the highest overlap (DSC 0.44), the lowest false discovery rate (0.55), and the smallest boundary error (HD_95%_ 7.6 mm). In comparison, the best reported threshold method (SUV > 4) performed worse (DSC 0.41, HD_95%_ 8.22 mm) [19^▪▪^].

Zhang *et al*.[20] compared contouring on [^18^F]F-PSMA-1007 and [^68^Ga]Ga-PSMA-11 PET/MRI. Fixed thresholds of 30–60% SUV_max_ systematically overestimated GTVs relative to mpMRI–derived volumes for both tracers, whereas manual PET contours did not substantially enlarge GTVs. The combined PET/MRI GTVs were, however, larger than mpMRI alone and had the highest concordance with histopathology in terms of tumour length.

Furthermore, Grefve *et al.* [15^▪^] observed that the fusion of contours manually delineated using both mpMRI and PSMA-PET resulted in better alignment with the histopathological lesion (ISUP GG≥4) regarding, lesion coverage (66%) and DSC (0.45), compared to adding a margin (1–3 mm) to mpMRI contours. However, mpMRI without added margin had a higher DSC (0.52) than PSMA-PET or mpMRI + PSMA-PET, reflecting tighter contours. Among the manual contouring approaches, the use of the STAPLE algorithm improved the lesion alignment [15^▪^,^21^].

Lastly, Geboers *et al.* [22^▪^] performed an analysis based on the detection of a quadrant containing csPCa and found that the combination of PSMA-PET and mpMRI had a higher sensitivity (80%) compared to only mpMRI (59%), or only PET (72%). The specificity was, however, lower for the combination (85%) compared to mpMRI (91%) or PET (92%).

Overall, fixed SUV thresholds lack generalisability across tracers and yield poorer spatial overlap compared to manual delineation. Furthermore, the integration of PSMA-PET with mpMRI may improve lesion coverage and better spare normal tissue compared to single-modality approaches.

### Interobserver variability in MRI- and PET-based gross tumour volume delineation

Interobserver variability in contouring was consistently lower when incorporating PSMA-PET, either alone or in combination with mpMRI, compared with mpMRI alone [15^▪^,^20^,22^▪^]. The study by Zarei *et al.* [19^▪▪^] used [^68^Ga]Ga-PSMA-11 PET/MRI followed by whole-mount histopathology after radical prostatectomy to assess contours made by four experienced radiation oncologists. Moderate agreement (DSC = 0.68) and the lowest false discovery rate was observed when using the STAPLE algorithm, compared to various semi-automatic approaches.

Zhang *et al.* [20] extended these findings in a cohort with contours made using [^18^F]F-PSMA-1007 PET/MRI or [^68^Ga]Ga-PSMA-11 PET/MRI, and reported comparable interreader consistency and DSC values exceeding 0.7 for both tracers using manual delineation. Furthermore, they found that combined PET/mpMRI-defined GTVs tended to be larger and had higher overlap between contours (DSC of 0.91 – 0.96), compared to PET alone (0.82 – 0.90) or mpMRI alone (0.82 – 0.90).

In a quadrant-based analysis, Geboers *et al.* [22^▪^] reported higher agreement with PSMA-PET regarding the presence of csPCa (84%) than mpMRI (74%), suggesting that PSMA-PET might be less observer-dependent and easier to reproduce.

### Application of artificial intelligence for tumour delineation in multiparametric MRI and prostate-specific membrane antigen-PET

Delineating intraprostatic lesions is labour-intensive and subject to considerable interobserver variability, motivating research into artificial intelligence based contouring using mpMRI and/or PSMA-PET. Tsui *et al.* [23^▪▪^] trained a segmentation model (89 patients) and generated 30 DIL, which were compared to five radiation oncologist contours. Comparable DSCs were observed between the artificial intelligence generated and radiation oncologist contours, mirroring the interobserver variability between the radiation oncologists. The artificial intelligence-contours also yielded dose escalation plans, which were similar to those produced by the majority of radiation oncologists. However, the artificial intelligence-model resulted in 26.9% false-positives, significantly smaller lesions, and had a lower lesion-specific sensitivity than the radiation oncologists.

Kostyszyn *et al.* [24] trained a network on 142 treatment naïve primary PCa patients imaged with [^68^Ga]Ga-PSMA-11 PET/CT and validated it on an internal ([^68^Ga]Ga-PSMA-PET and [^18^F]F-PSMA-1007 PET, in *n* = 18 and *n* = 19, respectively) and external patient cohort ([^68^Ga]Ga-PSMA-11 PET, *n* = 18). The median DSC between artificial intelligence-contours and expert GTVs were 0.84, 0.81, and 0.83, respectively. Ghezzo *et al.* [25] performed an external validation of this network on the intraprostatic lesions of patients imaged with [^68^Ga]Ga-PSMA-11 PET/CT (*n* = 39) or PET/MRI (*n* = 46). The model demonstrated good agreement with contours delineated by two expert readers (median DSC = 0.74) on both PET/CT and PET/MRI platforms, and performed best in larger lesion. This was evidenced by a positive correlation between expert-defined GTV volume and DSC.

More recently, Glemser *et al.* [26^▪^] integrated a deep-learning PI-RADS equivalent into combined [^18^F]F-PSMA-1007 PET/MRI, reporting good agreement (mean DSC of 0.44) between artificial intelligence and expert contours. However, this study was performed on only a small heterogeneous cohort of seven patients with localised or advanced disease, and validation in a larger external population is warranted.

## DISEASE OUTCOME AND TOXICITY

The use of focal boost volumes targeting intraprostatic lesions raises ongoing questions about potential increases in toxicity. Furthermore, median GTV target volumes can vary across imaging modalities [27,28^▪^]. This section examines the toxicity profile and disease outcomes of focal boost radiotherapy, in which the GTV has been defined using mpMRI alone or in combination with PSMA-PET.

## TOXICITY

The FLAME trial and one arm of the DELINEATE trial used standard fractionation (95 Gy in 35 fractions and 82 Gy in 37 fractions, respectively) with mpMRI-defined GTVs, and additional 2 mm margin to the GTV in the DELINEATE trial [6,7]. Both trials reported low rates of acute genitourinary and gastrointestinal toxicity.

Moderate or extreme hypofractionation to mpMRI-GTVs (60 Gy/20 fractions and/or 50 Gy/5 fractions to the GTV) was used in the hypo-FLAME, hypo-FLAME 2.0, arm B of the DELINEATE trial, and the studies of Cloitre *et al.* [30^▪^] and Tsurugai *et al.* [29][6,31,32]. These studies reported promising rates of acute gastrointestinal and genitourinary toxicity. For instance, among these studies only one case of grade 4 GI toxicity was observed in the moderately hypofractionated arm of the DELINEATE trial. In addition, Camden *et al.* [33^▪^] evaluated a sequential boost regimen (45 Gy/25 fractions to the prostate followed by a boost of 18 Gy/3 fractions) and found no unacceptable levels (CTCAE > 2) of acute gastrointestinal or genitourinary toxicity.

In the HypoFocal study, 25 patients received up to 75 Gy (20 fractions) in the boosted region defined by the union of PSMA-PET and mpMRI-derived GTVs (union-GTV) [27]. For the PROBE study the union-GTV received 40 Gy (five fractions) and the area with overlapping GTV-PET and GTV-MRI (GTV-overlap) was treated with 42 Gy (five fractions) [28^▪^]. Both studies showed promising acute toxicity profiles, with no grade 3 genitourinary and GI toxicities observed at follow-up of 6 months or 90 days or less.

In addition to acute adverse events, late toxicity was found to be within acceptable ranges in studies using standard and (moderately) hypofractionated doses. Late grade 3 genitourinary and gastrointestinal toxicities remained low across all cohorts, with rates of 3% genitourinary and 6% gastrointestinal at 2-year follow-up in the Cloitre *et al.* [30^▪^] study, 2% genitourinary and 1% gastrointestinal at 5-year follow-up in the HYPO-FLAME trial, and 2% genitourinary and 0% gastrointestinal at 18-month follow-up in the Camden *et al.* study [30^▪^,33^▪^,^34^,35^▪^]. The HypoFocal study, which targeted union-GTVs, reported 2-year follow-up toxicity outcomes of 24% for grade ≥2 genitourinary toxicity and 8% for gastrointestinal toxicity [36^▪▪^]. During follow-up, two (8%) patients experienced grade 3 toxicity; however, these events were considered to be of multifactorial origin.

Taken together, these findings indicate that PSMA-PET/MRI–guided focal boost radiotherapy is well tolerated. However, ongoing and future long-term follow-up studies are required to confirm this.

## DISEASE OUTCOME AND RECURRENCE PATTERNS

The FLAME trial demonstrated that standard fractionated focal boost radiotherapy to the intraprostatic GTV significantly improved 5-year biochemical disease-free survival (bDFS), local failure-free survival, metastasis-free survival, and overall disease-free survival compared with standard treatment [5]. Similarly, (moderately) hypofractionated regimens targeting mpMRI-GTVs achieved high rates of bDFS in the DELINEATE trial (arm A: 98.2%, B: 96.7%, C: 95.1%, 5-year follow-up), the Hypo-FLAME trial (93%, 5-year follow-up), and in the Camden *et al.* [33^▪^] cohort (95.3%, 3-year follow-up) [6,34,35^▪^]. The HypoFocal study targeting mpMRI/PSMA-PET-GTVs observed a bDFS rate of 96% at 35-month follow-up with only one patient developing multiocular metastatic disease, possibly due to undetected initial spread [36^▪▪^].

Further analysis of recurrence patterns after focal boost radiotherapy to mpMRI-GTVs was conducted by Menne Guricova *et al.* [37^▪^] and by Cloitre *et al.* [30^▪^]. Menne Guricova *et al.* [37^▪^] analysed intraprostatic recurrence patterns and found that 96% of failures occurred within the primary GTV, with a median time to failure of 4.8 years. Notably, the patients who recurred had received a lower mean focal boost dose (76.5 Gy) compared to the cohort median D98% dose of 84 Gy.

Cloitre *et al.* [30^▪^] investigated patients that did not receive androgen deprivation therapy (ADT) after hypofractionated focal boost treatment. At median follow-up of 82-months, 21% of patients had intra-prostatic recurrence, and of these recurrences, 57.1% showed full or partial overlap with the GTV. Although DIL recurrence was infrequent, a notable proportion of patients developed extraprostatic disease. Lastly, Camden *et al.* [33^▪^] reported no local or nodal recurrences at 3 years of follow-up.

## DISCUSSION AND FUTURE PERSPECTIVES

Collectively, the histopathology-validated studies reviewed demonstrate that the integration of mpMRI and PSMA-PET markedly enhances the accuracy of intraprostatic lesion delineation, improving both spatial concordance and alignment with histological boundaries compared to mpMRI alone or with margin expansion (Table 1). Among PSMA-PET–based approaches, manual contouring remained the most consistent and reliable method, particularly when supported by consensus algorithms such as STAPLE [15^▪^,^19^^▪▪^,^21^]. By contrast, threshold-based segmentation techniques exhibited lower accuracy and limited generalisability across different PSMA tracers.

Furthermore, combining mpMRI with PSMA-PET improved the detection of intraprostatic lesions, particularly for clinically relevant volumes. However, this increased sensitivity came at the cost of reduced specificity, reflected in higher false discovery rates and lower spatial accuracy. The use of combined imaging also tended to result in larger GTVs, as observed in the HypoFocal clinical trial, though this was not associated with a notable increase in acute treatment-related toxicity [27].

A meta-analysis conducted by Dhar *et al.* [38^▪▪^] evaluated the performance of mpMRI and/or PET in intraprostatic lesion delineation in patients treated with radical prostatectomy, including studies published up to August 2022. The pooled sensitivity for the detection of quadrants with csPCa, for mpMRI, PSMA-PET, and the combination of both was 64.7% (*n* = 13), 75.7% (*n* = 12), and 70.3% (*n* = 5), respectively. Consistent with findings from the studies reviewed here, PSMA-PET and PSMA-PET-mpMRI outperformed mpMRI in terms of sensitivity. Furthermore, this study also observed a loss in specificity for PSMA-PET-mpMRI (81.9%) compared to PSMA-PET (87.1%) or mpMRI (86.4%).

To summarise, these studies support the use of a hybrid imaging as the most reliable approach for delineating DILs in the context of focal boost radiotherapy. However, several limitations should be considered when interpreting these findings. For instance, most studies included relatively small patient cohorts and, with few exceptions, were retrospective in design, factors that limit the generalisability and robustness of the conclusions [15^▪^,^19^^▪▪^,^20^,22^▪^]. Additionally, while spatial-overlap metrics (DSC, lesion coverage) were widely reported, distance-based measures (HD_95%_, MDA) were used infrequently. Without systematic boundary-based evaluation, the accuracy of the GTVs remains incompletely characterised. Lastly, all included studies relied on rigid registration of whole-mount histopathology to in-vivo imaging. This type of alignment leads to in-plane errors, which can influence the spatial-overlap and distance-based metrics [39].

PSMA-PET has demonstrated modestly greater interobserver consistency than mpMRI, although variability remains a challenge across all imaging modalities. Standardised semi-automatic approaches, such as fixed SUV thresholding, have shown limited generalisability across different PSMA tracers and consistently underperformed compared to expert contours when validated against histopathology.

Emerging artificial intelligence based segmentation tools and adaptive thresholding techniques show promise in accelerating delineation and reducing variability. Nonetheless, these methods often misestimate lesion boundaries and lack the reliability required for unsupervised clinical use. While artificial intelligence driven contours exhibit consistency levels comparable to expert observers, they remain limited by higher false positives and reduced sensitivity. Therefore, the most robust approach for GTV definition in focal boost radiotherapy remains the integration of PET and MRI, guided by expert manual editing and consensus algorithms to optimise lesion coverage while sparing adjacent normal tissue.

Reported rates of acute and late toxicity following standard or moderately hypofractionated focal boost radiotherapy have generally been low. Comparable toxicity profiles were observed between treatments targeting mpMRI-defined GTVs and combined mpMRI–PSMA-PET-defined GTVs, even when larger boost volumes were used. However, this review did not account for heterogeneity in dose constraints, fractionation schedules, image-guidance protocols, or bladder and rectal preparation techniques, all of which may influence both toxicity and oncological outcomes.

The long-term toxicity and clinical outcome data for PSMA-PET–defined boost volumes are limited to a single small cohort (HypoFocal; *n* = 25) with a median follow-up of 35 months [36^▪▪^]. A definitive conclusion regarding disease outcomes requires longer follow-up.

The long-term follow-up studies using MRI-based-GTV delineation suggest that focal-boost techniques generally achieve durable intraprostatic control. Recurrences most frequently occur in regions that either received subtherapeutic doses within the planned GTV or in adjacent untreated tissue, underscoring the dual necessity of precise lesion delineation and robust dose delivery [30^▪^,37^▪^].

## CONCLUSION

Manual delineation of GTVs using combined mpMRI and PSMA-PET imaging improves lesion detection and demonstrates greater concordance with histopathological intraprostatic lesions. Although PET/MRI-defined GTVs are generally larger than those delineated using MRI alone, hypofractionated focal boost treatments targeting these volumes have demonstrated acceptable acute toxicity profiles. Focal boost to MRI-defined GTVs improves oncological outcomes with manageable toxicity; however, whether targeting PET/MRI-defined GTVs offers additional clinical benefit warrants further investigation.

## Acknowledgements

*None.*

### Financial support and sponsorship

*This work is financially supported by the Hanarth Fonds Fund.*

### Conflicts of interest

*None related to this work.*

## Supplementary Material

Supplemental Digital Content

## REFERENCES AND RECOMMENDED READING

Papers of particular interest, published within the annual period of review, have been highlighted as:

▪ of special interest
▪▪ of outstanding interest
