# Supplementary material for: Integration of prostate-specific membrane antigen-PET and multiparametric MRI for gross tumour volume definition in localised and locally advanced prostate cancer treated with image-guided radiotherapy
Source: Curr Opin Urol. 2025 Jul 15;35(5):554–61. doi: 10.1097/MOU.0000000000001321 (PMC12337896; doi:10.1097/MOU.0000000000001321)
Supplement: Supplemental Digital Content [file couro-35-554-s001.docx]

Supplementary File 1 – search strategy

**Search strategy and selection criteria**A nonsystematic literature search was conducted on the first of April 2025 to retrieve records published since 2023 concerning intraprostatic GTV delineation in primary localised prostate cancer and focal boost radiotherapy. Articles were retrieved using MEDLINE via PubMed and the top 100 hits from the Google Scholar search engine (Supplementary File 1). Additional citations were identified through backward citation searches and recommendations from co-authors. The results were presented as a narrative review.

**1. MEDLINE via PubMed**

((Gross tumour volume[TIAB]) AND (Dominant intraprostatic lesion[TIAB])) AND (delineation[TIAB]) AND ((primary prostate cancer[TIAB]) AND (PSMA-PET[TIAB]) OR (magnetic resonance imaging [TIAB]) OR (MRI[TIAB]) OR (Artificial intelligence [TIAB]) OR (AI[TIAB]))

**2. Google Scholar search engine**

“Gross tumour volume”|“Dominant intraprostatic lesion” AND “delineation|contouring” AND “PSMA-PET”|“MRI”|“AI”
